# SUV_mean_ on baseline [^18^F]PSMA-1007 PET and clinical parameters are associated with survival in prostate cancer patients scheduled for [^177^Lu]Lu-PSMA I&T

**DOI:** 10.1007/s00259-023-06281-6

**Published:** 2023-06-05

**Authors:** Philipp E. Hartrampf, Thomas Hüttmann, Anna Katharina Seitz, Hubert Kübler, Sebastian E. Serfling, Wiebke Schlötelburg, Kerstin Michalski, Steven P. Rowe, Martin G. Pomper, Andreas K. Buck, Uta Eberlein, Rudolf A. Werner

**Affiliations:** 1 Department of Nuclear Medicine, University Hospital Würzburg, Würzburg, Germany; 2 Department of Urology and Paediatric Urology, University Hospital Würzburg, Würzburg, Germany; 3 The Russell H Morgan Department of Radiology and Radiological Science, Johns Hopkins School of Medicine, Baltimore, MD USA

**Keywords:** PSMA, Prostate cancer, [^177^Lu]Lu-PSMA I&T, Radioligand therapy, Overall survival, [^18^F]PSMA-1007, Theranostics

## Abstract

**Background:**

Quantification of [^68^ Ga]-labeled PSMA PET predicts response in patients with prostate cancer (PC) who undergo PSMA-targeted radioligand therapy (RLT). Given the increasing use [^18^F]-labeled radiotracers, we aimed to determine whether the uptake derived from [^18^F]PSMA-1007 PET can also identify responders and to assess its prognostic value relative to established clinical parameters.

**Methods:**

We retrospectively analyzed 103 patients with metastatic, castration-resistant PC who were treated with [^177^Lu]Lu-PSMA I&T. We calculated SUV_mean_, SUV_max_, PSMA-avid tumor volume (TV), and total lesion PSMA (defined as PSMA-TV*SUV_mean_) on pre-therapeutic [^18^F]PSMA-1007 PET. Laboratory values for hemoglobin, C-reactive protein (CRP), lactate dehydrogenase (LDH), aspartate aminotransferase (AST), and alkaline phosphatase (AP) were also collected prior to RLT. We performed univariable Cox regression followed by multivariable and Kaplan–Meier analyses with overall survival (OS) serving as endpoint. Last, we also computed a risk factor (RF) model including all items reaching significance on multivariable analysis to determine whether an increasing number of RFs can improve risk stratification.

**Results:**

A total of 48 patients died and median OS was 16 months. On univariable Cox regression, SUV_mean_, CRP, LDH, hemoglobin, and the presence of liver metastases were significantly associated with OS. On multivariable Cox regression, the following significant prognostic factors for OS were identified: SUV_mean_ (per unit, HR, 0.91; *P* = 0.04), the presence of liver metastases (HR, 2.37; *P* = 0.03), CRP (per mg/dl, HR, 1.13; *P* = 0.003), and hemoglobin (per g/dl, HR, 0.76; *P* < 0.01). Kaplan–Meier analysis showed significant separation between patients with a SUV_mean_ below or above a median SUV_mean_ of 9.4 (9 vs 19 months, HR 0.57; *P* = 0.03). Of note, patients with only one RF (median OS not reached) showed longest survival compared to patients with two (11 months; HR 2.43 95% CI 1.07–5.49, *P* = 0.02) or more than two RFs (7 months; HR 3.37 95% CI 1.62–7.03, *P* < 0.001).

**Conclusion:**

A lower SUV_mean_ derived from [^18^F]PSMA-1007, higher CRP, lower hemoglobin, and the presence of liver metastases are associated with reduced OS in patients undergoing RLT. An early RF model also demonstrated that an increasing number of those factors is linked to worse outcome, thereby emphasizing the importance of clinical and imaging parameters for adequate risk stratification.

**Supplementary Information:**

The online version contains supplementary material available at 10.1007/s00259-023-06281-6.

## Introduction

Prostate-specific membrane antigen (PSMA)-targeted radioligand therapies (RLT) using [^177^Lu]Lu-PSMA-617 have been shown to improve overall survival (OS) and/or biochemical response (as measured by prostate specific antigen (PSA) levels) compared to standard of care and cabazitaxel [1, 2]. This led to approval by the U.S. Food and Drug Administration [3] and the European Medicines Agency [4]. However, not all patients respond to RLT, highlighting the need for reliable prognosticators of treatment outcomes, preferably by combining available clinical and imaging parameters at baseline.

Several studies have identified clinical prognostic factors for patients receiving [^177^Lu]Lu-PSMA-617, including the presence of visceral metastases [5-8], elevated alkaline phosphatase (AP) [9, 10], C-reactive protein (CRP) levels [11], prior chemotherapy [8, 12, 14], elevated lactate dehydrogenase (LDH) [9-11, 14], elevated aspartate aminotransferase (AST) [6, 14], and lower hemoglobin [6, 11]. For survival prediction in patients treated with [^177^Lu]Lu-PSMA I&T, Heck et al. also identified the presence of visceral metastases and a rising LDH as being associated with OS [5], while a recent analysis from our group showed that baseline CRP, LDH, AST, and time interval until RLT initiation were associated with survival in patients receiving the identical compound [14].

Beyond those clinical parameters, recent efforts also turned towards investigating baseline PSMA PET uptake in men treated with PSMA-directed RLT. For instance, Seifert et al. reported on PSMA-positive tumor volume (PSMA-TV) [14] and PSMA total lesion quotient derived from [^68^ Ga]Ga-PSMA-11 as being associated with OS [14], while a low average SUV_max_ of all PSMA-positive lesions was a negative prognosticator for survival [14]. Widjaja and coworkers also reported that quantification of [^68^ Ga]Ga-PSMA-11 was prognostic for PSA response in subjects being treated with [^177^Lu]Lu-PSMA-617 [22]. In addition, the TheraP trial also used [^68^ Ga]Ga-PSMA-11 and sites of disease with a SUV_mean_ ≥ 10 had a higher likelihood of favorable response to [^177^Lu]Lu-PSMA-617 [14]. All of those previous studies, however, focused on the prognostic value of [^68^ Ga]-labeled PSMA PET in the context of [^177^Lu]Lu-PSMA-617, while the prognostic capability of [^18^F]-labeled PSMA PET agents in men treated with other PSMA-targeted β-emitters has not been elucidated yet in a larger cohort. Such information, however, may be of importance, as recent years have witnessed an expanded use and clinical adoption of [^18^F]-labeled PSMA agents, in particular in the theranostic context [22].

As such, in this study, we aimed to identify prognostic baseline imaging parameters using [^18^F]PSMA-1007 in patients who were treated with [^177^Lu]Lu-PSMA I&T. Additionally, we aimed to provide a risk factor (RF) model that includes not only imaging but also clinical parameters available at the time of treatment planning. Such an approach may help to better identify patients at high risk for treatment failure of PSMA RLT.

## Material and methods

### Patient cohort

This single-center study analyzed 103 patients with metastatic, castration-resistant PC (mCRPC) treated with [^177^Lu]Lu-PSMA I&T who had all received pre-therapeutic imaging using [^18^F]PSMA-1007 PET/CT. All subjects gave written informed consent and the local Ethics Committee waived the need for further approval due to the retrospective character of the study (waiver no. 20220502 01). Parts of this cohort have already been reported in [14, 14, 27, 27, 29], but without using imaging derived parameters for predicting early PSA response and OS.

### Pre-therapy workup

All patients received whole body [^18^F]PSMA-1007 PET scans with contrast-enhanced diagnostic CT for attenuation correction and anatomical registration prior to initiation of RLT. Median injected activity was 301 MBq (251–417 MBq). Time between PSMA PET/CT and initiation of RLT was in median 29 days (0–118 days). Detailed description of the synthesis of the radiotracer and imaging procedures are also available in [22].

On admission day prior to RLT, pre-treatment blood samples were taken for serum chemistry (PSA level, creatinine, LDH, AST, AP, and CRP) and routine hematology (leukocytes, hemoglobin, platelets) using EDTA tubes (Sarstedt, Nuembrecht, Germany). The samples were examined using an automated analyzer (Sysmex XN-9000, Kobe, Japan) for hematology and a fully automated modular analyzer (Roche Cobas, Basel, Switzerland) for serum chemistry [29]. Medical records were reviewed for patient history.

### Image analysis

PET/CT images were analyzed using Syngo.via (Syngo.via; VB60, Siemens Healthineers, Erlangen, Germany) software for visual interpretation and the Beth Israel plugin for FIJI (ImageJ) for segmentation [14]. Lesions with visually higher PSMA uptake compared to background were considered positive for local recurrence or metastases, excluding non-malignant structures (e.g., salivary glands and celiac ganglia [29]). SUV_max_, SUV_peak_, and SUV_mean_ of the total of all lesions as well as whole body PSMA positive tumor volume (PSMA-TV) were determined using semi-automatic analysis with a fixed threshold SUV of 3. Thereafter, an expert PSMA PET reader (S.E.S.) conducted review of all lesions, with a second review by another expert reader (R.A.W.) in inconclusive cases. Whole body total lesion PSMA (TL-PSMA) was calculated by multiplying PSMA-TV by SUV_mean_ [22].

### Treatment protocol

We performed standard procedures for RLT. This included synthesizing [^177^Lu]Lu-PSMA I&T, as outlined in [14]. Patients received 6.0 GBq of [^177^Lu]Lu-PSMA I&T every 6 to 8 weeks, with a maximum of 8 cycles. In case of impaired kidney function, activity was reduced by approximately 20%.

### Statistical analysis

Statistical analysis was done using GraphPad Prism 9.3.0 on Windows (GraphPad Software, San Diego, CA). Median and range are presented. Uni- and multi-variable logistic regressions were used to identify baseline imaging and clinical parameters that predict early PSA response. Early PSA response was defined as any PSA decrease 8 weeks after the first cycle. OS was defined as the time from first cycle to death (presented as median). For survival prediction, uni- and multi-variable cox regressions were used (with outlier correction). Kaplan–Meier curves and log-rank tests were also applied using median values as cutoffs. Cox regression models for OS were compared applying a null model (without any parameter) using the Akaike information criterion (AIC) [35] and Harrell’s C-statistic [14]. Lower AIC and higher Harrell’s C values are indicators for a better-fit model [14, 35]. Hazard ratio (HR) and 95% confidence interval (95% CI) are displayed. Finally, we also computed an RF model including all items reaching significance on multivariable analyses. A *P*-value < 0.05 was considered statistically significant.

## Results

### Patients’ characteristics

A total of 103 patients with a median initial Gleason score of 9 (6–10) and a median age of 71 years (46–88 years) were included. A median time interval between initial diagnosis and 1st cycle of RLT (interval_Diagnosis-RLT_) of 62 (range, 9–274) months was recorded and patients were treated with a median of three cycles [^177^Lu]Lu-PSMA I&T (median cumulative activity, 14.4 GBq; range, 4.8–50.9 GBq). Forty-eight patients died and median OS was 16 months. Detailed patients characteristics are displayed in Table 1. Therapy was well tolerated and a brief overview of adverse events can be found in Supplemental Table 1.

**Table 1:** Patient’s characteristics

*Clinical variables*	Median (range)
Age at first cycle of PSMA RLT (years)	71 (46–88)
Interval_Diagnosis-RLT_ (months)	62 (9–274)
Treatment cycles per patient	3 (1–8)
Cumulative activity (GBq)	14.4 (4.8–50.9)
Gleason score	9 (6–10)
*Baseline laboratory values*	
PSA (ng/ml)	159 (2.9–3590)
CRP (mg/dl)	0.75 (0.01–29.3)
LDH (U/l)	268.5 (152–1800)
Hemoglobin (g/dl)	11.8 (6.0–14.8)
AST (U/l)	29.0 (13.8–546.7)
AP (U/l)	146.0 (31.0–5818)
*Baseline PET parameters*	
PSMA-TV (cm^3^)	546.7 (7.9–3820)
TL-PSMA (cm^3^)	4998 (51.7–32,065)
SUV_max_	63.4 (13.2–424.5)
SUV_peak_	33.3 (8.5–181.6)
SUV_mean_	9.4 (4.3–23.7)
Sites of metastases	**n (%)**
Bone	101 (98.1)
Lymph node	71 (68.9)
Visceral	38 (36.9)
Liver	22 (21.4)
Lung	14 (13.6)
*Prior treatments*	**%**
Radical prostatectomy	40.8
Primary radiation therapy to the prostate	11.7
Antihormonal treatment	100
Enzalutamide	70.9
Abiraterone	70.9
Chemotherapy	77.7

### *Baseline SUV*_*mean*_* and age at first cycle are independently associated with early PSA response after 8 weeks*

On univariable analysis, SUV_mean_ (per unit, HR 1.18, 95% CI 1.06–1.33; *P* = 0.004), LDH (per unit, HR 0.99, 95% CI 0.99–0.99; *P* = 0.01), age at first cycle (per year, HR 1.07, 95% CI 1.01–1.14; *P* = 0.02), interval_Diagnosis-RLT_ (per month, HR 1.01, 95% CI 1.00–1.02; *P* = 0.02), CRP (per unit, HR 0.86, 95% CI 0.74–0.97; *P* = 0.03), and hemoglobin (per unit, HR 1.30, 95% CI 1.02–1.70; *P* = 0.04) were significantly associated with PSA response (Table 2). For multivariable cox regression, we included SUV_mean_, LDH, CRP, hemoglobin and age at first cycle. Only SUV_mean_ (per unit, HR 1.18, 95% CI 1.05–1.35; *P* = 0.008) and age (per year, HR 1.07, 95% CI 1.01–1.14; *P* = 0.04) remained significant (Table 2).

**Table 2:** Uni- and multi-variable logistic regression model for early PSA response

	Univariable			Multivariable		
	OR	95% CI	*P*-value	OR	95% CI	*P*-value
PSMA-TV (cm^3^)	1.00	1.00–1.00	0.77			
TL-PSMA (cm^3^)	1.00	1.00–1.00	0.18			
SUV_Mean_	1.18	1.06–1.33	0.004	1.18	1.05–1.35	0.008
SUV_Peak_	1.02	1.00–1.04	0.08			
SUV_Max_	1.00	1.00–1.01	0.27			
visceral metastases	0.97	0.42–2.26	0.95			
liver metastases	0.80	0.28–2.24	0.67			
PSA µg/l	1.00	1.00–1.00	0.08			
CRP mg/dl	0.86	0.74–0.97	0.03	0.97	0.82–1.12	0.71
LDH (37 °C U/l)	0.995	0.99–0.999	0.01	0.99	0.99–0.999	0.07
Hemoglobin g/dl	1.30	1.02–1.70	0.04	1.13	0.83–1.55	0.45
AST (37 °C U/l)	0.99	0.98–1.01	0.51			
AP (37 °C U/l)	1.00	0.99–1.00	0.81			
Age at 1st cycle	1.07	1.01–1.14	0.02	1.07	1.00–1.14	0.04
Time period between initial diagnosis and 1st RLT (months)	1.01	1.00–1.02	0.02			
Prior CTx	0.51	0.18–1.36	0.18			
Gleason	0.75	0.47–1.18	0.22			

*OR* odds ratio, *CI* confidence interval, *PSMA-TV* PSMA-positive tumor volume, *TL-PSMA* total lesion PSMA, *SUV* standardized uptake value, *PSA* prostate-specific antigen, *CRP* C-reactive protein, *LDH* lactate dehydrogenase, *AST* aspartate aminotransferase*, AP* alkaline phosphatase, *Interval*_*Diagnosis-RLT*_ time period between initial diagnosis and 1st RLT, *CTx* chemotherapy. Significant values are marked in bold

### *Baseline SUV*_*mean*_*, CRP, hemoglobin, and the presence of liver metastases are independently associated with survival*

On univariable analysis, CRP (per unit, HR 1.15, 95% CI 1.08–1.23), LDH (per unit, HR 1.003, 95% CI 1.001–1.004), hemoglobin (per unit, HR 0.65, 95% CI 0.53–0.79; *P* < 0.001, each), the presence of liver metastases (HR 2.73, 95% CI 1.28–5.49; *P* = 0.006), and SUV_mean_ (per unit, HR 0.91, 95% CI 0.84–0.99; *P* < 0.05) were significantly associated with OS (Table 3). However, the largest changes of the models relative to the reference standard (“null model,” AIC: 254.8) were observed for hemoglobin (AIC, 237.4, C-index: 0.70), followed by CRP (AIC, 241.0, C-index: 0.75), and LDH (AIC, 244.7, C-index: 0.72). SUV_mean_, however, demonstrated higher AIC (251.5) and lower C-index (0.66), thereby indicating a less prominent role for this SUV metric relative to the other independent clinical parameters (Table 3).

**Table 3:** Univariable cox regression model for overall survival

	HR	95% CI	AIC	Harrell’s C	*P*-value
Null model			254.8		
PSMA-TV (cm^3^)	1.00	1.00–1.00	255.8	0.61	0.31
TL-PSMA (cm^3^)	1.00	1.00–1.00	256.8	0.54	0.94
SUV_mean_	0.91	0.84–0.99	251.5	0.66	0.03
SUV_peak_	0.99	0.97–1.01	255.2	0.64	0.22
SUV_max_	0.99	0.99–1.00	256.7	0.63	0.71
Visceral metastases	1.84	0.98–3.43	253.2	0.59	0.05
Liver metastases	2.73	1.28–5.49	250.4	0.59	0.006
PSA µg/l	1.00	0.99–1.00	255.2	0.60	0.17
CRP mg/dl	1.15	1.08–1.23	241.0	0.75	< 0.0001
LDH (37 °C U/l)	1.003	1.001–1.004	244.7	0.72	< 0.0001
Hemoglobin g/dl	0.65	0.53–0.79	237.4	0.70	< 0.0001
AST (37 °C U/l)	1.01	0.99–1.02	254.1	0.65	0.08
AP (37 °C U/l)	1.00	0.99–1.00	256.7	0.65	0.80
Age at 1st cycle	0.99	0.96–1.04	256.8	0.57	0.90
Time period between initial diagnosis and 1st RLT (months)	1.00	0.99–1.00	254.7	0.63	0.17
Prior CTx	1.02	0.53–2.06	256.8	0.52	0.96
Gleason*	1.11	0.77–1.61		0.55	0.59

*HR* hazard ratio, *CI* confidence interval, *AIC* Akaike information criterion, *PSMA-TV* PSMA-positive tumor volume, *TL-PSMA* total lesion PSMA, *SUV* standardized uptake value, *PSA* prostate-specific antigen, *CRP* C-reactive protein, *LDH* = lactate dehydrogenase, *AST* aspartate aminotransferase*, AP* alkaline phosphatase, *Interval*_*Diagnosis-RLT*_ time period between initial diagnosis and 1st RLT*, CTx* chemotherapy. Significant values are marked in bold. Lower AIC and higher Harrell’s C values indicate a better-fit model [14, 35]

^*^Establishing a null-model was not possible due to missing values

Multivariable Cox regression analysis then revealed that CRP (per unit, HR 1.13, 95% CI 1.04–1.22; *P* = 0.003), hemoglobin (per unit, HR 0.76, 95% CI 0.61–0.93; *P* = 0.007), the presence of liver metastases (HR 2.37, 95% CI 1.05–5.13; *P* = 0.03), and SUV_mean_ (per unit, HR 0.91, 95% CI 0.83–0.99; *P* < 0.05) remained significant. LDH (per unit, HR 1.001, 95% CI 1.000–1.003), however, failed to reach significance (*P* = 0.10; Table 4).

**Table 4:** Multivariable cox regression model for overall survival

	HR	95% CI	AIC	Harrell’s C	*P*-value
Null model			254.8		
SUV_Mean_	0.91	0.83–0.99	224.3	0.81	0.04
liver metastases	2.37	1.05–5.13			0.03
CRP mg/dl	1.13	1.04–1.22			0.003
LDH (37 °C U/l)	1.00	1.00–1.00			0.10
Hemoglobin g/dl	0.76	0.61–0.93			0.007

*HR* hazard ratio, *CI* confidence interval, *AIC* Akaike information criterion, *SUV* Standardized uptake value, *CRP* C-reactive protein, *LDH* lactate dehydrogenase. Significant values are marked in bold

### *Baseline SUV*_*mean*_*, CRP, hemoglobin, and the absence/presence of liver metastases differentiate between responders vs. non-responders*

**Fig. 1:**
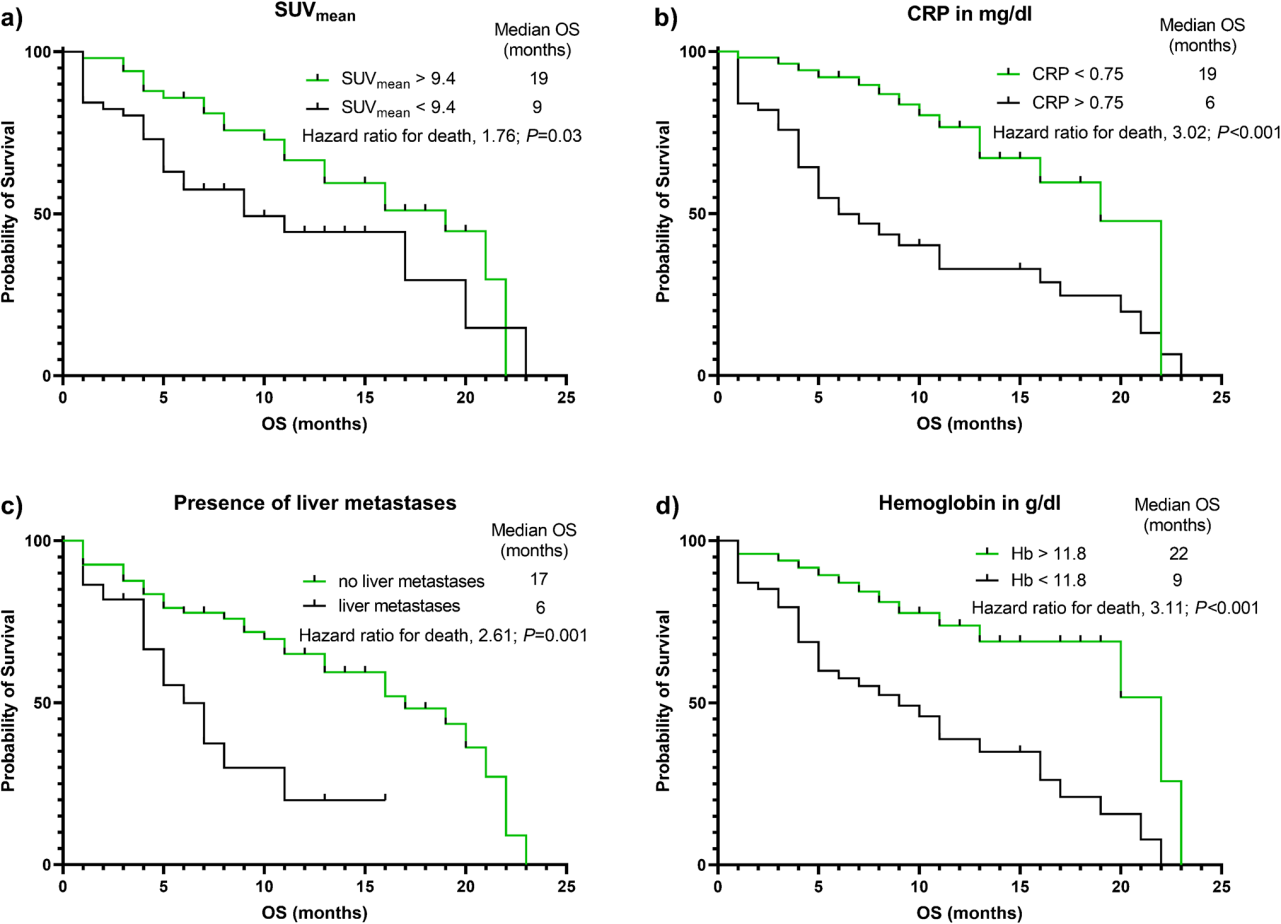
Kaplan–Meier analyses of patients grouped according to median SUV_mean_, C-reactive protein (CRP), and hemoglobin (Hb) and the absence or presence of liver metastases. **a** Patients with higher baseline SUV_mean_ (19 vs. 9 months; HR 1.76 95% CI 0.98–3.17, *p* = 0.03), **b** with lower baseline CRP (19 vs. 6 months; HR 3.02 95% CI 1.71–5.34, *p* < 0.001), **c** without liver metastases (17 vs. 6 months; HR 2.61 95% CI 1.14–5.94, *p* = 0.001), and **d** with higher baseline hemoglobin (22 vs. 9 months; HR 3.11 95% CI 1.76–5.50, *p* < 0.001) showed longer overall survival (OS)

**Fig. 2:**
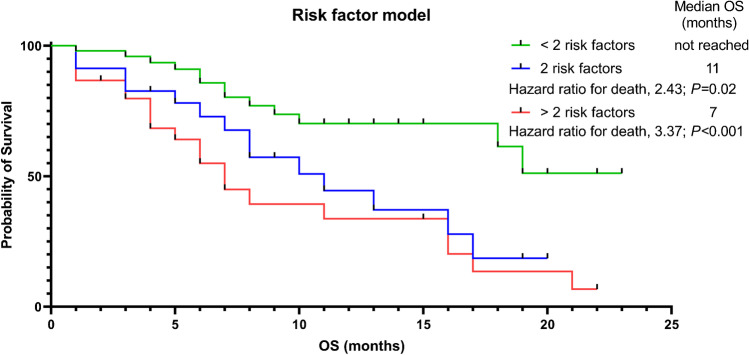
Risk stratification model including all clinical and imaging parameters reaching significance on multivariate analysis. Lower SUV_mean_, higher C-reactive protein, lower hemoglobin, and the presence of liver metastases each reflect an individual risk factor (RF). Patients with one RF showed longest overall survival (OS) compared to patients with exactly two or more than two RFs, with the latter subgroup exhibiting shortest survival

Patients were then stratified according to the median values of SUV_mean_, CRP, and hemoglobin as well as the absence/presence of liver metastases. Kaplan–Meier analyses showed longer OS in patients with lower baseline CRP (19 vs. 6 months; HR 3.02 95% CI 1.71–5.34), higher hemoglobin levels (22 vs. 9 months; HR 3.11 95% CI 1.76–5.50; *P* < 0.001, each), and in patients without liver metastases (17 vs. 6 months; HR 2.61 95% CI 1.14–5.94, *P* = 0.001; Fig. 1). Last, higher baseline SUV_mean_ was also associated with prolonged OS (19 vs. 9 months; HR 1.76 95% CI 0.98–3.17, *P* = 0.03). Those clinical and imaging parameters were then also applied to RF modeling, with lower SUV_mean_, higher CRP, lower hemoglobin, and the presence of liver metastases each reflecting an RF. Patients with only one RF (median OS not reached) showed longest survival compared to patients with two (11 months; HR 2.43 95% CI 1.07–5.49, *P* = 0.02) or more than two RFs (7 months; HR 3.37 95% CI 1.62–7.03, *P* < 0.001; Fig. 2).

**Fig. 3:**
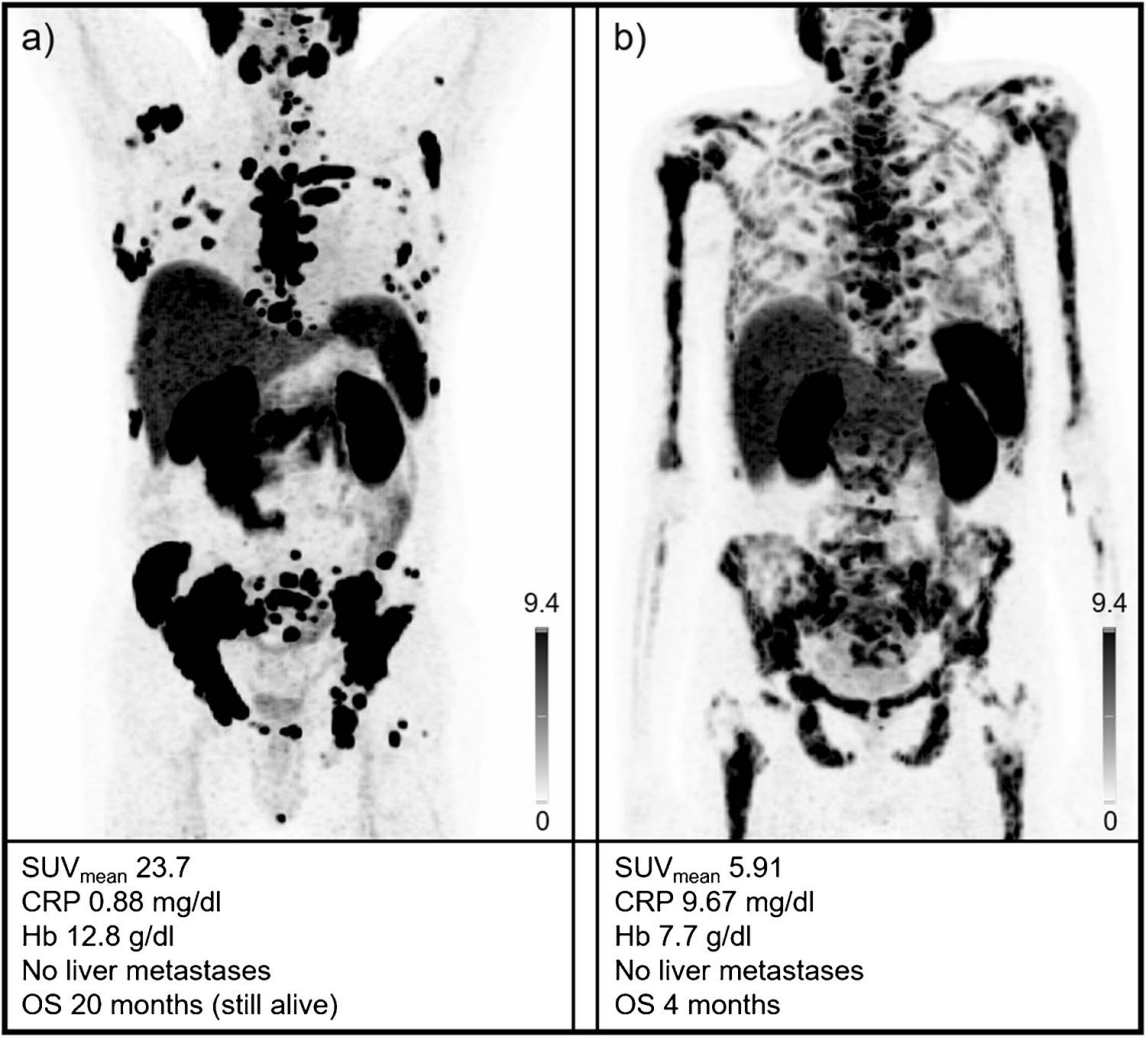
Baseline [^18^F]PSMA-1007 PET of two patients with different outcomes after radioligand therapy with [^177^Lu]Lu-PSMA I&T. Maximum intensity projections are displayed. The baseline values of the different parameters and the maximum intensity projections are also provided. **a** This patient showed high tracer uptake and no risk factors apart from slightly elevated CRP levels. **b** This patient exhibited only moderate tracer uptake with a patchy pattern, elevated CRP and reduced hemoglobin, resulting in three risk factors. Overall survival of the patient provided in (**a**) was significantly longer (20 months at date of censoring versus patient in (**b**) with 4 months). SUV = standardized uptake value, CRP = C-reactive protein, Hb = hemoglobin, OS = overall survival

Figure 3 shows examples of two patients with different outcomes along with respective risk factors for each case.

## Discussion

In 103 patients with mCRPC who were treated with [^177^Lu]Lu-PSMA I&T, lower baseline SUV_mean_ derived from [^18^F]PSMA-1007 PET and lower age at the 1st cycle of RLT were associated with early PSA failure after 8 weeks. Furthermore, lower baseline SUV_mean_, higher CRP, decreased hemoglobin levels, and hepatic metastases were independently associated with reduced OS. Last, when computing an RF model, the presence of more than two of those factors also identified patients with shortest survival, thereby demonstrating that all available clinical and imaging parameters should be taken into account to identify men at highest risk for RLT failure.

Regarding clinical parameters available at baseline, the vast majority of studies published to date have focused on the use of [^177^Lu]Lu-PSMA-617, showing that CRP, LDH, liver enzymes, or AP can identify men prone to treatment failure [6, 10, 11, 14, 29]. In the present analysis, however, only patients treated with another commonly used β-emitting PSMA radiotherapeutic (i.e., [^177^Lu]Lu-PSMA I&T) were included. In this regard, we have already reported that baseline CRP is a significant predictor in PCa patients treated with this compound [14], which has also been described for [^177^Lu]Lu-PSMA-617 [6, 11, 14]. As a major drawback, however, CRP is nonspecific and can be significantly elevated in inflammatory disease [22].

Comparable to our previous and other reports, we could not confirm a prognostic ability of elevated baseline AP for OS after RLT with [^177^Lu]Lu-PSMA I&T [5, 14, 35], although this was reported for [^177^Lu]Lu-PSMA-617 [6, 9, 10, 12, 29]. Interestingly, in our study, age at initiation of RLT was significantly associated with early PSA response but not OS. This has also been previously reported by Widjaja et al. using [^177^Lu]Lu-PSMA-617 [22]. This phenomenon may be partially explained by the assumption that older patients may have less aggressive variants of underlying tumor biology [14] and, thus, are more likely to respond to PSMA-targeted RLT, irrespective of the agent.

Taken together, although survival has been demonstrated to be comparable for both compounds [22], outcome prediction based on clinical parameters may be complex for both PSMA-targeted compounds. As such, extrapolation from [^177^Lu]Lu-PSMA I&T to those subjects scheduled for therapy with -617 should be made with caution. Thus, we also investigated the prognostic value of an [^18^F]-labeled PSMA PET compound for identifying patients with high risk of treatment failure, while previous studies mainly focused on [^68^ Ga]-labeled PET compounds. Those considerations are further fueled by the fact that there is an increasing shift towards radiofluorine in the context of PSMA-targeted molecular imaging and risk stratification in a theranostic approach [22], thereby making our findings relevant for the broader nuclear medicine community.

We observed that the presence of PSMA-avid hepatic liver lesions has a negative impact on survival, a finding that has also been extensively described for [^177^Lu]Lu-PSMA-617-treated patients imaged with [^68^ Ga]-labeled radiotracers [5-8]. On a quantitative assessment, an increasing SUV_mean_ was associated with a higher probability of early PSA response and longer OS. Those results are in line with TheraP, which showed that men with a PSMA SUV_mean_ ≥ 10 in their metastatic lesions had a higher likelihood of favorable PSA response to [^177^Lu]Lu-PSMA-617 when compared to chemotherapy [14]. The analysis of the pre-therapeutic [^68^ Ga]-labeled PSMA PET/CT scans from the VISION trial also showed that a higher SUV_mean_ was associated with an improved survival [39]. Of note, our analysis showed an impact on survival, and patients with SUV_mean_ > 9.4 (which is virtually identical to the cut-off used in TheraP) showed a median OS twice as long as patients with SUV_mean_ below this cut-off. The SUV_mean_ might thus be seen as a marker for heterogeneity of PSMA expression, as a low value could indicate a partially decreased/absent PSMA expression of the tumor cells. In preclinical work, it has been shown that the fraction of PSMA-positive cells correlates with PSMA RLT efficacy and that an overall decreased PSMA expression is associated with a weaker effect of RLT [29].

The present study also provides results on PET quantification using [^18^F]PSMA-1007 in a large and homogenously treated cohort of 103 patients with mCRPC. A recent study including 20 subjects investigated the use of this radiotracer for identifying high-risk patients along with DNA damage response markers, and the authors reported that SUV_max_ failed to predict progression-free survival. Those differences relative to the present study may be explained by the larger number of individuals investigated in our analysis and by varying endpoints (progression-free vs. OS) [22]. Moreover, the presented AIC and Harrell’s C indices used in our report provide an assessment of the different fitted survival models, thereby allowing a determination as to which parameter has the most prominent role in outcome. In this regard, CRP and hemoglobin yielded improved statistics (reflected by lower AIC and higher C-index) when compared to SUV_mean_, suggesting a greater importance of clinical parameters when compared to PET-based quantification. Nonetheless, the herein provided RF model still showed that a combination of all available (clinical and PET) parameters should be taken into account to identify those patients with shortest survival. In this regard, the herein used RF model is easy to implement in clinical practice, with more than two RFs tightly linked to decreasing survival. This is also in line with a recent study providing nomograms, which also incorporated varying clinical predictors (including hemoglobin) and tumor burden derived from [^68^ Ga]Ga-PSMA-11 PET [7]. Taken together, those previous findings on [^68^ Ga]-,and our results on [^18^F]-labeled, PSMA PET further emphasize the importance of considering both clinical and imaging parameters prior to treatment with RLT.

## Conclusions

Lower SUV_mean_ derived from [^18^F]PSMA-1007 PET is associated with less favorable PSA response rates and overall survival in patients treated with [^177^Lu]Lu-PSMA I&T. Furthermore, higher CRP, lower hemoglobin levels, and the presence of hepatic metastases were independently associated with reduced OS. Finally, a RF model including all of those parameters also demonstrated that an increasing number of those factors is linked to worse outcome, in particular in patients with more than two RFs. As such, our results may also emphasize the importance of a holistic approach to treatment planning, which should include clinical and imaging parameters for adequate risk stratification.

### Supplementary information

Below is the link to the electronic supplementary material.

Supplementary file1 (DOCX 20 KB)

## Data Availability

The main data presented in this study are available in the article. Detailed information about the image analysis or the overall survivals of the subjects presented in this study are available on request from the corresponding author.
