# Supplementary material for: SUVmean on baseline [18F]PSMA-1007 PET and clinical parameters are associated with survival in prostate cancer patients scheduled for [177Lu]Lu-PSMA I&T
Source: Eur J Nucl Med Mol Imaging. 2023 Jun 5;50(11):3465–74. doi: 10.1007/s00259-023-06281-6 (PMC10542708; doi:10.1007/s00259-023-06281-6)
Supplement: Supplementary file 1 — Supplementary file1 (DOCX 20 KB) [file 259_2023_6281_MOESM1_ESM.docx]

**Supplemental Table 1: Classifications according to CTCAE version 5.0 for leukocytes, hemoglobin, platelets and eGFR for patients treated with [^177^Lu]Lu-PSMA I&T at baseline and after the last available cycle.** "New toxicity" describes whether a new adverse event has occurred during therapy. No grade III/IV toxicities occurred under therapy.

|  |  | Grade 0 | Grade I | Grade II | Grade III | Grade IV |
| --- | --- | --- | --- | --- | --- | --- |
| leukocytes | baseline | 89/103 (86.4%) | 12/103 (11.7%) | 2/103  (1.9%) | 0 | 0 |
|  | last cycle | 53/84 (63.1%) | 29/84 (34.5%) | 2/84  (2.4%) | 0 | 0 |
|  | new toxicity |  | 23 | 2 | 0 | 0 |
|  | | | | | | |
| hemoglobin | baseline | 5/103 (4.9%) | 80/103 (77.7%) | 15/103 (14.6%) | 3/103 (2.9%) | 0 |
|  | last cycle | 2/84  (2.4%) | 60/84 (71.4%) | 22/84 (26.2%) | 0 | 0 |
|  | new toxicity |  | 4 | 14 | 0 | 0 |
|  | | | | | | |
| platelets | baseline | 96/103 (93.2%) | 6/103  (5.8%) | 1/103  (1%) | 0 | 0 |
|  | last cycle | 70/84 (83.3%) | 12/84 (14.3%) | 2/84  (2.4%) | 0 | 0 |
|  | new toxicity |  | 8 | 1 | 0 | 0 |
|  | | | | | | |
| eGFR | baseline | 44/103 (42.7%) | 47/103 (45.6%) | 11/103 (10.7%) | 1/103 (1.0%) | 0 |
|  | last cycle | 31/84 (36.9%) | 37/84 (44.0%) | 16/84 (19.0%) | 0 | 0 |
|  | new toxicity |  | 7 | 7 | 0 | 0 |
